# Rethinking intercurrent events in defining estimands for tuberculosis trials

**DOI:** 10.1177/17407745221103853

**Published:** 2022-07-19

**Authors:** Tra My Pham, Conor D Tweed, James R Carpenter, Brennan C Kahan, Andrew J Nunn, Angela M Crook, Hanif Esmail, Ruth Goodall, Patrick PJ Phillips, Ian R White

**Affiliations:** 1 MRC Clinical Trials Unit at UCL, London, UK; 2 Department of Medical Statistics, London School of Hygiene and Tropical Medicine, London, UK; 3 UCSF Center for Tuberculosis, University of California San Francisco, San Francisco, CA, USA

**Keywords:** Tuberculosis, estimand, intercurrent events, intention to treat, per protocol

## Abstract

**Background/aims:**

Tuberculosis remains one of the leading causes of death from an infectious disease globally. Both choices of outcome definitions and approaches to handling events happening post-randomisation can change the treatment effect being estimated, but these are often inconsistently described, thus inhibiting clear interpretation and comparison across trials.

**Methods:**

Starting from the ICH E9(R1) addendum’s definition of an estimand, we use our experience of conducting large Phase III tuberculosis treatment trials and our understanding of the estimand framework to identify the key decisions regarding how different event types are handled in the primary outcome definition, and the important points that should be considered in making such decisions. A key issue is the handling of intercurrent (i.e. post-randomisation) events (ICEs) which affect interpretation of or preclude measurement of the intended final outcome. We consider common ICEs including treatment changes and treatment extension, poor adherence to randomised treatment, re-infection with a new strain of tuberculosis which is different from the original infection, and death. We use two completed tuberculosis trials (REMoxTB and STREAM Stage 1) as illustrative examples. These trials tested non-inferiority of new tuberculosis treatment regimens versus a control regimen. The primary outcome was a binary composite endpoint, ‘favourable’ or ‘unfavourable’, which was constructed from several components.

**Results:**

We propose the following improvements in handling the above-mentioned ICEs and loss to follow-up (a post-randomisation event that is not in itself an ICE). First, changes to allocated regimens should not necessarily be viewed as an unfavourable outcome; from the patient perspective, the potential harms associated with a change in the regimen should instead be directly quantified. Second, handling poor adherence to randomised treatment using a per-protocol analysis does not necessarily target a clear estimand; instead, it would be desirable to develop ways to estimate the treatment effects more relevant to programmatic settings. Third, re-infection with a new strain of tuberculosis could be handled with different strategies, depending on whether the outcome of interest is the ability to attain culture negativity from infection with any strain of tuberculosis, or specifically the presenting strain of tuberculosis. Fourth, where possible, death could be separated into tuberculosis-related and non-tuberculosis-related and handled using appropriate strategies. Finally, although some losses to follow-up would result in early treatment discontinuation, patients lost to follow-up before the end of the trial should not always be classified as having an unfavourable outcome. Instead, loss to follow-up should be separated from not completing the treatment, which is an ICE and may be considered as an unfavourable outcome.

**Conclusion:**

The estimand framework clarifies many issues in tuberculosis trials but also challenges trialists to justify and improve their outcome definitions. Future trialists should consider all the above points in defining their outcomes.

## Introduction

### Tuberculosis treatment

Tuberculosis (TB) is one of the leading causes of death from an infectious disease globally, with an estimated 10 million people developing active disease and an estimated 1.4 million deaths in 2019.^1^

Highly effective short-course treatments of 6 months for drug-susceptible TB based on regimens containing isoniazid and rifampicin were established in the 1970s and 1980s,^2,3^ but shortening regimens below 6 months has only happened very recently.^4^ In contrast, the World Health Organization^5^ recommends an all-oral bedaquiline-containing regimen of 9–12 months duration for patients with confirmed multidrug- or rifampicin-resistant TB. There is very limited trial evidence for treating rifampicin-resistant TB, although several trials are now being conducted.^6–10^

Late-phase trials have investigated the efficacy and safety of novel drug-susceptible TB and rifampicin-resistant TB treatment regimens. However, results in programmatic settings (i.e. as implemented by the National TB Programmes) are often inferior to results in trials. Under trial conditions, standard TB therapy for treating drug-susceptible pulmonary TB cures over 90% of participants;^11^ in programmatic settings, this falls to approximately 80%–85%.^1^ Cure rates of treatment for rifampicin-resistant TB are typically only 50%–60% in programmatic settings.^1^

The long duration and side effects of TB treatments are associated with poor adherence in programmatic conditions, which is then associated with a higher risk of failure (i.e. failure to achieve culture conversion), development of mycobacterial resistance, and relapse after stopping treatment. Efforts to curb the TB epidemic require treatments that are shorter, less toxic, and simpler to deliver.

### Motivation for using the estimand frameworkin TB trials

It is common in TB trials that the primary outcome is binary, usually ‘favourable’ or ‘unfavourable’. Recent trials have used a composite endpoint constructed from several components, such as presence or absence of culture conversion, subsequent relapse, and changes of treatment due to adverse events.^10–12^ These components reflect different aspects of the regimens under comparison over the entire course of treatment and follow-up. In previous trials, some components were inevitably included as ‘unfavourable’, because (1) the actual subsequent outcome was missing and ‘unfavourable’ was believed to be the most likely outcome and (2) the chosen method of analysis required a binary classification, although this rationale was not often well articulated.

A new and fruitful way of thinking about the outcome in TB trials uses the estimand framework, which describes all aspects of the treatment effect that a trial aims to estimate. The ICH E9(R1) addendum recently provided a structured framework for defining estimands and sensitivity analyses in clinical trials;^13^ it has been adopted by most leading regulatory authorities. The addendum emphasises the importance of aligning the target of estimation, method of estimation, and sensitivity analysis for a given trial objective.

Discussions on the estimand framework have started in the context of TB trials following the introduction of the addendum.^14–16^ Many aspects of constructing the estimand have already been addressed in the way previous TB trials defined the outcome. Nevertheless, the lack of a formal adoption of the estimand language in the design and analysis of these trials makes it important to assess, in a systematic way, whether all relevant intercurrent (i.e. post-randomisation) events (ICEs) which affect interpretation of or preclude measurement of the intended final outcome, have been considered, and whether they have been handled appropriately. This lack of clarity also means that it is difficult to identify precisely what the trial aims to estimate.

Most TB trials conducted in recent years have been of non-inferiority design in which both modified intention-to-treat and per-protocol analyses are performed, and non-inferiority is typically declared if results are consistent across these two analyses. However, modified intention-to-treat and per-protocol analyses handle ICEs differently, and consequently, they target different estimands. It is therefore not clear how conclusions about non-inferiority should be made in the presence of discordance between the two analyses. More importantly, it is not always clear which estimand is targeted by the per-protocol analysis approach of excluding patients with certain ICEs,^17^ so even if non-inferiority is demonstrated it does not necessarily mean that the intervention really is non-inferior for a reasonable estimand/research question. Shifting focus from two methods of analysis (modified intention-to-treat and per-protocol) to the estimand framework can also stimulate development of improved analysis methods.

### Aims and objectives

Our aim is to identify and address the potential issues to be considered in constructing estimands for future TB trials, with a particular focus on the handling of ICEs. We hope to demonstrate that this approach will improve the ability of future TB trials to address relevant clinical questions.

## Methods

Starting from the ICH E9(R1) addendum’s definition of an estimand, we use our experience of conducting large Phase III TB treatment trials and our understanding of the estimand framework to identify the key decisions regarding how different event types are handled in the primary outcome definition, and the important points that should be considered in making such decisions. We focus on the objective of evaluating the programmatic effect of novel TB treatment regimens, that is, the effect if the regimens being evaluated were introduced as the standard of care. We consider this effect from the perspectives (1) of the patient and (2) of the healthcare provider.

Two of our previous Phase III TB trials, REMoxTB^11^ and STREAM Stage 1,^6,7,10^ evaluating short regimens for drug-susceptible and rifampicin-resistant TB, respectively, are used for illustration. These trials tested non-inferiority of new TB treatment regimens compared with a control regimen. The primary outcome was a binary composite endpoint, ‘favourable’ or ‘unfavourable’, which was constructed from several components.

An estimand is made up of five attributes: (1) treatment; (2) population; (3) outcome; (4) ICEs and how they are handled; and (5) population-level summary. These attributes are described for REMoxTB and STREAM Stage 1 in Table 1. The addendum discussed five general strategies for handling ICEs when defining the clinical question: (1) treatment policy; (2) composite; (3) hypothetical; (4) principal stratum; and (5) while on treatment (Table 2).^13^ Each of these strategies can be used alone, or in combination with others for handling different types of ICEs.

**Table 1.** Estimand attributes in REMoxTB and STREAM Stage 1.

Estimand attribute	Definition	REMoxTB (*N* = 1931)	STREAM Stage 1 (*N* = 424)
1. Treatment regimens to be compared	The treatment condition of interest and, as appropriate, the alternative treatment condition to which comparison will be made	Two moxifloxacin-containing 4-month regimens versus the standard 6-month control regimen	A shorter experimental regimen with allowable treatment extension under certain circumstances (9–11 months) versus a long standard-of-care regimen consistent with 2011 WHO recommendations (20+ months)
2. Patient population of interest	The population of patients targeted by the clinical question	Adult patients with drug-susceptible pulmonary TB	Adult patients with rifampicin-resistant pulmonary TB
3. Outcome definition	The variable (or endpoint) to be obtained for each patient that is required to address the clinical question	A composite favourable outcome defined as patients with a negative culture status at 18 months (at or after 72 weeks), who had not already been classified as having an unfavourable outcome, and whose last positive culture result was followed by at least two negative culture results	A composite favourable outcome defined as patients with a sputum culture-negative status at 132 weeks, with no intervening positive culture or previous unfavourable status which included changes for adverse events
4. Intercurrent events	Events occurring after treatment initiation that affect either the interpretation or the existence of the outcome associated with the clinical question of interest	1. Treatment changes (including both minor and major changes) and treatment extension. 2. Poor adherence to randomised treatment. 3. Re-infection with a new strain of TB. 4. Death (both related and unrelated to TB).	
5. Population-level summary	A summary measure providing a basis for comparison between treatment conditions	The difference in proportions of patients with a favourable outcome betweentreatment regimens	

TB: tuberculosis; WHO: World Health Organization.

**Table 2.** Strategies for handling ICEs.

Strategy	Definition
1. Treatment policy	The occurrence of the ICE (e.g. a change of treatment) is considered as part of the randomised treatment. This strategy cannot be implemented for ICEs that are terminal events (e.g. death), since values of the outcome after the occurrence of the ICE do not exist
2. Composite	The occurrence of the ICE is incorporated into the endpoint as an additional component, alongside other measures of the clinical outcome (e.g. an unfavourable outcome due to positive culture results or death related to TB)
3. Hypothetical	The endpoint is evaluated in a hypothetical scenario in which the ICE did not occur (e.g. if the patient had not had a change of treatment)
4. Principal stratum	The stratum of trial participants in which the ICE would not occur regardless of randomised treatments is identified and taken as the target population (e.g. in patients who would not have a change of treatment regardless of which regimen they were randomised to receive)
5. While on treatment	The endpoint is evaluated based on data prior to the occurrence of the ICE (e.g. before the patient had a change of treatment)

TB: tuberculosis; ICE: intercurrent event.

We consider four common ICEs that are relevant to REMoxTB and STREAM Stage 1 and can complicate the task of defining the estimand: (1) treatment changes (both minor and major) and treatment extension; (2) poor adherence to randomised treatment; (3) re-infection with a new strain of TB; and (4) death (both TB- and non-TB-related). Strategies for handling these ICEs are discussed from the patient and healthcare provider perspectives, taking into account both statistical and clinical considerations. We acknowledge that other objectives (and perspectives) may require other estimands to be defined, potentially with different strategies employed for handling the same ICEs.^18,19^ It is not our intention to provide prescriptive guidance on how the ICEs considered in this article should always be handled. This is because the handling of ICEs will depend on the context of the trials and the exact research questions addressed by the trials, as well as the perspectives of stakeholders.

The next section discusses the handling of some common ICEs as well as loss to follow-up (a post-randomisation event that is not in itself an ICE^13^).

## Results

### Handling of some common ICEs

Table 3 summarises our suggested strategies for handling four common ICEs in the two example trials. The frequency of each of these four ICEs in REMoxTB and STREAM Stage 1 is reported in Tables 4 and 5, using the trial publications^10,11^ and data obtained from the Platform for Aggregations of Clinical TB Studies (TB-PACTS)^20^ and the STREAM trial team, respectively. The TB-PACTS initiative is a public–private partnership launched in May 2015 by Critical Path Institute (C-Path), the Special Programme for Research and Training in Tropical Diseases, the Global Alliance for TB Drug Development (TB Alliance), and St. George’s, University of London. The purpose of this exercise was to see how well these trials had already aligned with the aspect of handling ICEs under the estimand framework. Depending on how frequently certain ICEs occur, choosing different approaches for dealing with them might also lead to more noticeable changes in the trial results.

**Table 3.** Current handling of ICEs in REMoxTB and STREAM Stage 1 and proposed strategies following the estimand framework.

ICE	What is it?	Why is it an ICE?	How has it been handled in REMoxTB and STREAM Stage 1?	How might it be handled following theestimand framework?	
				Patient perspective	Healthcare providerperspective
Treatment changes andtreatment extension	Change of one or more drugs withinthe regimen; switch regimen; extendduration of treatment	Data collected after a treatment change are no longer reflective of the patient’s status whilereceiving the original treatment	Unfavourable composite strategy for a change of one drug for REMoxTB; treatment policy strategy for a change of up to two drugs for STREAM Stage 1; unfavourable composite strategy for a change of two or more drugs for STREAM Stage 1; treatment policy strategy for treatment extension of a certain length to make up for missed doses	Treatment policy, with longer duration and greater toxicity as secondary outcomes; hypothetical	Composite; treatmentpolicy; hypothetical
Poor adherence	Failure to take the required doses of adrug; failure to take certain drugs in theregimen; failure to complete theduration of treatment	The regimens being compared at the end of thetrial are not the same as the ones being randomised topatients at the start of the trial (assuming that treatmentregimen is ‘fixed’)	Per-protocol analysis, including only patients taking ≥80% of doses	Hypothetical; treatment policy	Hypothetical
Re-infection	Patients are infected with a strain of TB that isdifferent from the originally infected one	The occurrence of re-infection with a different strain ofTB hinders our ability to confirm whether the patient hasbeen cured from their original strain of TB	Re-infections are classified as not assessable (e.g. missing data) and excluded from analysis	Treatment policy	Treatment policy;hypothetical
Death	Death, related or unrelated to TB	For those who die before completing the trial, theirclinical data and culture results no longer exist and aretruncated by death	Unfavourable composite strategy for patients dying from any cause during the treatment phase, except from violent or accidental cause (e.g. road traffic accident), not including suicide, or patients dying from TB-related cause during the follow-up phase in REMoxTB; unfavourable composite strategy for patients dying from any cause in STREAM Stage 1	Composite	Composite; hypothetical

TB: tuberculosis; ICE: intercurrent event.

**Table 4.** Frequency and the handling of each ICE in REMoxTB (based on Gillespie et al.^11^ and data obtained from TB-PACTS^20^).

ICE^a^		Treatment regimen				How ICE was handledin the analysis	
		C	I	E	Total	mITT	PP
		*N* = 640	*N* = 655	*N* = 636	*N* = 1931		
Treatment changes andtreatment extension^b^	Adverse reaction (treatment phase)	18	15	9	42	Unfavourable	Excluded
	Withdrawal of consent(treatment phase)	8	18	8	34	Unfavourable	Excluded
	Other investigatordecision(treatment phase)	2	5	0	7	Unfavourable	Excluded
	Retreated for TB(follow-up)	14	18	27	59	Unfavourable	Unfavourable(*N* = 58); excluded(*N* = 1,inadequate treatment)
	Total	42	56	44	142		
Poor adherence	Relocation(treatment phase)	2	4	4	10	Unfavourable	Excluded
	No completion of treatment(treatment phase)	13	10	6	29	Unfavourable	Excluded
	Inadequate treatment	0	2	0	2	Unfavourable	Excluded
	Total	15	16	10	41		
Re-infection		10	13	19	42	Excluded	Excluded
Death	Non-TB death(follow-up)	4	3	0	7	Excluded	Excluded
	Non-violent death (treatment phase)	5	6	7	18	Unfavourable	Unfavourable
	Death from TB or respiratorydistress (follow-up)	2	0	0	2	Unfavourable	Unfavourable
	Total	11	9	7	27		

C: control; I: isoniazid group; E: ethambutol group; mITT: modified intention to treat; PP: per protocol; TB: tuberculosis; ICE: intercurrent event.

a To avoid double counting, we defined intercurrent events following the order of exclusion from the analysis as presented in the publication flow chart.

b Treatment changes do not include restart of treatment following relapse after culture-negative status during follow-up.

**Table 5.** Frequency and the handling of each ICE in STREAM Stage 1 (based on Nunn et al.^10^ and data obtained from the STREAM Stage 1 trial team).

ICE^a^		Treatment regimen			How ICE was handedin the analysis	
		L	S	Total	mITT	PP
		*N* = 142	*N* = 282	*N* = 424		
Treatment changes and extension:started two or more additional drugs^b^	Never achieved culture conversion	0	1	1	Unfavourable	Unfavourable
	Bacteriological reversion on treatment	2	9	11	Unfavourable	Unfavourable
	Reversion on treatment with limited bacteriology	1	0	1	Unfavourable	Unfavourable
	Investigator decision	2	0	2	Unfavourable	Unfavourable
	Participant withdrew consent for treatment	0	3	3	Unfavourable	Unfavourable
	Treatment change after adverse event	2	1	3	Unfavourable	Unfavourable
	Total	7	14	21		
Treatment changes and extension: extended treatmentbeyond that allowed	Treatment extension after adverse event	0	2	2	Unfavourable	Unfavourable
	Treatment extension after poor adherence or loss to follow-up	0	1	1	Unfavourable	Unfavourable
	Total	0	3	3		
Poor adherence	Inadequate treatment	43	19	62	Favourable (*N* = 39);unfavourable (*N* = 20);not assessable (*N* = 3)^c^	Excluded
Re-infection		1	6	7	Excluded	Excluded
Death	Never achieved culture conversion	1	4	5	Unfavourable	Unfavourable
	Bacteriological reversion on treatment	1	1	2	Unfavourable	Unfavourable
	Culture positive when last seen	0	1	1	Unfavourable	Unfavourable
	Culture negative when last seen	5	9	14	Unfavourable	Unfavourable
	Total	7	15	22		

L: long regimen group; S: short regimen group; mITT: modified intention to treat; PP: per protocol; ICE: intercurrent event.

a To avoid double counting, we defined intercurrent events in the following order of exclusion from the mITT population: (1) did not complete an adequate course of treatment; (2) had re-infection.

b Treatment changes do not include changes following relapse after treatment (both bacteriological and with limited bacteriology).

c *N* = 20 ‘Unfavourable’ cases included *N* = 2 extended treatment beyond that allowed, *N* = 7 started two or more additional drugs, *N* = 11 no culture at or after 76 weeks; *N* = 3 ‘Not assessable’ cases included *N* = 1 re-infection, *N* = 2 lost to follow-up after 76 weeks, culture converted when last seen.

#### Treatment changes and treatment extension

Treatment changes include a change of one or more drugs within the assigned regimen or a wholesale switch to another regimen. Treatment changes are an ICE because data collected after a treatment change are no longer reflective of the patient’s status while receiving the original treatment.

Clinicians’ decisions to change treatment are typically based on treatment failure indicated by clinical assessment (e.g. weight loss, fevers, and night sweats) and/or microbiology (failure to attain/maintain culture negativity); patient inability to tolerate the treatment (including both minor, e.g. nausea, and major side effects, e.g. liver dysfunction, neuropathy, and loss of vision); and poor adherence to treatment. Criteria for treatment changes in the trial setting may differ from those under programmatic conditions when less information is available (e.g. microbiological samples are collected less frequently). Fewer treatment options may also be available in a national TB programme.

In REMoxTB, patients with a single drug change to their regimen were classified as having an unfavourable outcome, that is, this ICE was handled by the composite strategy. In STREAM Stage 1, one drug change was considered minor and permitted by the protocol, provided that the replacement drug was not a nitroimidazole or bedaquiline. Such minor changes were handled by the treatment policy strategy, that is, considered as part of the regimen. Other changes, such as the replacement of two or more drugs or the addition of a nitroimidazole or bedaquiline, were considered substantial and classified as an unfavourable outcome, that is, these ICEs were handled by the composite strategy.

Extensions to treatment duration up to a certain length to make up for missed treatment are usually allowed in the trial protocol (as was done in REMoxTB and STREAM Stage 1), indicating a treatment policy strategy.

From a patient perspective, we propose that a treatment change is not inherently a poor outcome. Nevertheless, treatment changes may predict later poor outcomes, because changing to a regimen of longer duration and/or greater toxicity may lead to worse adherence or to a less effective regimen. Therefore, the patients’ actual clinical outcome could be used rather than expecting the outcome to be unfavourable on the basis of treatment changes. This would suggest the treatment policy approach to this ICE. Then, secondary outcomes would capture the negative aspects of longer duration and greater toxicity.

Often, treatment changes are from the experimental regimen to the control regimen. If the trial’s aim is to evaluate the experimental regimen as a new standard of care with the current control regimen no longer freely available, then patients and healthcare providers would be interested in whether TB would be cured had the patients stayed on the same regimen. Hence, the hypothetical strategy could be used to handle this type of treatment change.

From the healthcare provider perspective, having a standardised regimen is easier to introduce and administer in practice. Changes to a regimen in the trial setting might be logistically demanding, expensive, or unavailable in practical settings with limited resources, meaning their use really is an unfavourable outcome for the programme. This would suggest the use of the composite strategy for handling treatment changes. Alternatives are to use the hypothetical strategy to evaluate the effectiveness of the originally assigned regimen, or (if changing to a less effective regimen might be a realistic treatment pathway in practice) to use the treatment policy strategy.

An experimental regimen achieving non-inferiority to the control regimen through extensive switches (e.g. switching to the control regimen) would not be considered desirable. The treatment policy strategy may potentially dilute any differences between regimens, increasing the chance of falsely declaring non-inferiority. In this context, the composite strategy might be appropriate, as was done in REMoxTB and STREAM Stage 1. If the control regimen is no longer available for switching in practice, then from the healthcare provider perspective, this ICE could be handled by the hypothetical strategy.

Risk of resistance in the community is another concern from the healthcare provider perspective. Since resistance cannot occur in cured patients, risk of resistance may reasonably be captured by the patient-perspective outcome. Alternatively, outcomes could be developed that specifically evaluate the risk of resistance.

#### Poor adherence to randomised treatment

The long duration, complexity, and side effects of TB treatments can affect the patient’s ability and willingness to fully adhere to the randomised regimen. Poor adherence can include either failure to take the required doses of a drug, or taking only certain drugs in the regimen, or failure to complete the duration of the assigned treatment; it is often a combination of all three reasons. The primary goal in the management of TB is to ensure that patients take their treatment as prescribed for the appropriate duration to ensure the highest chance of achieving a cure. Duration of therapy and toxicity are the major influences on patient adherence to medication.^21–23^ As with treatment changes, poor adherence to treatment is also an ICE.

Poor adherence to therapy also raises the risk of developing resistance.^24^ The development of drug resistance is associated with worsened treatment outcomes, so much clinical practice focuses on encouraging good adherence to treatment, for the sake of the patient’s personal outcome but also from a public health viewpoint.

Adherence is usually addressed in a per-protocol analysis, which includes only patients taking more than a certain level of the prescribed doses. In REMoxTB and STREAM Stage 1, adequate treatment was defined based on receipt of at least 80% of the assigned regimen within 120% of the intended duration. In both trials, the per-protocol analysis was performed simply excluding patients who did not receive an adequate amount of their allocated study regimen, potentially leaving non-comparable treatment groups.

We again consider defining the estimand from two perspectives. Patients might be interested in knowing how effective the treatment is in curing their TB if they follow the regimen carefully. Thus, the hypothetical strategy might be appropriate for handling poor adherence as an ICE. Patients might also be interested in the likelihood of being cured of TB as a consequence of attempting to take the treatment, in which case the treatment policy strategy, allowing poor adherence as part of the regimens, is appropriate. If a regimen’s toxicities are severe, not many patients will tolerate the regimen, so a hypothetical scenario of the treatment effects when patients continue with their regimen is of less practical interest. Thus, it may often be sensible to separate out non-adherence due to toxicity versus other reasons, and handle the former with the treatment policy strategy and the latter with the hypothetical strategy.

From the healthcare provider perspective, early treatment discontinuation is a strong indication of subsequent lack of cure which is an unfavourable outcome. It is therefore appropriate to handle this ICE using a composite strategy. Often TB trials use a non-inferiority design to compare new regimens that are less burdensome (e.g. shorter, simpler, and might be easier to tolerate) to the patients compared with the standard-of-care regimen. Per-protocol analysis was historically preferred to modified intention-to-treat analysis (although more recently this is less common), as non-adherence is usually anti-conservative when handled using the treatment policy strategy. Often, a per-protocol analysis is used as an attempt to estimate the treatment effect in a hypothetical setting in which all patients had completed a protocol-adherent course of treatment. Alongside this, healthcare providers are also interested in how the regimens perform in practice. Since treatment adherence in a trial is typically higher than in programmatic settings, one might instead compare the regimens in a hypothetical setting where adherence is poorer than in the trial conditions, thus matching the programmatic setting more closely.

#### Re-infection

This ICE is specific to TB trials (and potentially other infectious diseases such as COVID-19), and occurs when a patient is cured and subsequently re-infected with a new strain that is different from the original infection. Re-infection is an ICE because it hinders our ability to confirm whether the patient has been durably cured from their original strain of TB. In the presence of re-infection, the observed status of the patient at the end of the trial is not relevant to answer our clinical question of interest.

In REMoxTB and STREAM Stage 1, patients who were re-infected with a new strain of TB were classified as not assessable (i.e. missing data) and excluded from the analysis. From both the patient and healthcare provider perspectives, if the clinical question of interest is whether the patient is cured of any TB, then the treatment policy strategy could be used to handle re-infection, and the outcome of interest would be the ability to attain culture negativity from infection with any strain of TB. Alternatively, a treatment programme might be interested in successfully delivering a regimen where the patient is treated and cured of the presenting strain of TB, without ‘penalising’ the regimen for the patient being re-infected with a different strain of TB in an environment prone to re-infection. Therefore, considering the healthcare provider perspective, a hypothetical strategy could be used to evaluate the effect of treatment in a hypothetical scenario where re-infection did not occur.

#### Death

Death in TB trials may or may not be related to TB. If the ability to achieve cure in patients is assessed based on their clinical assessments and culture results, then for those who die before completing the trial, their clinical data and culture results no longer exist after death, that is, their data are truncated by death.^25–28^

Patients who die during the trial are often treated as having an unfavourable outcome, sometimes regardless of the cause of death such as in the case of STREAM Stage 1. This is appropriate if it is not possible to determine whether TB contributed to death. In REMoxTB, patients were classified as having an unfavourable outcome if they died from any cause during the 6-month treatment phase, except from violent or accidental cause (e.g. road traffic accident), but including suicide and TB-related causes. Patients dying from violent or accidental causes, or during the follow-up phase with no evidence of failure or relapse of their TB were classified as not assessable (i.e. missing data) and excluded from analysis.

Like re-infection, death (both TB- and non-TB-related) is intrinsically a poor outcome for the patient, and the composite strategy classifying patients who die as having an unfavourable outcome is appropriate. When the regimens are evaluated from the healthcare provider perspective, the same is true for TB-related death. For death not related to TB, in some plausible scenarios such as accidental death, patient outcomes could be compared across treatment arms in a hypothetical setting had the patients not died from such a cause.

### Handling loss to follow-up

The current recommendation is not to view loss to follow-up, study withdrawal, or other forms of missing data (e.g. administrative censoring in trials with survival outcomes) as ICEs. The rationale for this is that events such as loss to follow-up do not – of themselves – change the interpretation of the outcome; they only result in the outcome being unobserved. Handling loss to follow-up is therefore an analysis problem in which suitable inferences are drawn about the complete (observed and unobserved) data.^29–31^

However, in TB trials, some losses to follow-up result in early discontinuation of treatment (i.e. an ICE). For this reason, loss to follow-up has often been handled like an ICE.^10–12^

In STREAM Stage 1, patients who did not have a culture result within the week 76 analysis window (up to 14 days either side of the scheduled week 76 date) because of loss to follow-up were handled as follows. For patients lost to follow-up in the standard-of-care treatment arm (with duration necessarily exceeding 76 weeks), loss to follow-up was regarded as early discontinuation of treatment and consequent lack of cure, so they were classified as having an unfavourable outcome. Patients lost to follow-up in the experimental treatment arm (with duration substantially less than 76 weeks) were handled in the same way in order to reduce bias. Similarly, in REMoxTB, patients lost to follow-up or withdrawn from the study before the 6-month visit (i.e. before the end of the randomised treatments) were classified as having an unfavourable outcome, even though the last 2 months of treatment for those on experimental arms merely comprised placebo. Therefore, this type of early loss to follow-up was implicitly considered as an ICE in both trials.

We propose conceptually separating loss to follow-up (which is a missing data issue and not an ICE) from treatment discontinuation (which is an ICE), while recognising that they may coincide. Loss to follow-up with treatment discontinuation would then be handled as appropriate, for example, using a composite strategy, classifying patients who discontinued treatment early as having an unfavourable outcome. Loss to follow-up without treatment discontinuation would be handled as missing patient data and could be imputed based on observed data from patients who also stopped receiving their randomised treatment prematurely but remained in the trial. For patients who were lost to follow-up having completed the treatment phase, their missing outcome data could also be imputed from the observed data. Treatment status after loss to follow-up might also be unknown, in which case the occurrence of the treatment discontinuation ICE would itself be missing data that could be imputed.

## Discussion

This article is a first attempt to comprehensively outline the process of handling ICEs as part of defining estimands in trials of TB treatment regimens, based on guidance from the ICE E9(R1) addendum. In such a fast-moving area, it is important to have clear specification of precisely what is being estimated, in order to allow new treatment regimens to be efficiently investigated. Proper specification of the estimand and the handling of ICEs can help ensure study objectives are clear, and that the design and analysis are aligned with the trials’ research objectives. The exact estimand will depend on the trials’ specific research objectives, and so it is impossible to offer prescriptive advice on which strategies to use. However, our aim in this article is to provide trialists with a framework to help them identify which estimands are most appropriate for their own trials.

Working out the frequency of each ICE for REMoxTB and STREAM Stage 1 from published data several years after the trials were completed was challenging, since patients could have experienced more than one ICE and the ordering of the ICEs was not always clear from the trial publications. This exercise highlighted the problem in trying to identify ICEs and creating alternative estimands after a trial has been published (without clean raw data and input from the trial team). This emphasises why the process of identifying ICEs and selecting strategies for handling them should be pre-specified at the design stage of the trial, alongside other attributes of the estimand.

Changing the strategies for handling the ICEs (and subsequently, the estimation methods that follow) could potentially change the results and conclusions of the trial.^32^ The extent of this change would depend on factors such as how common the ICEs were, whether the occurrences of the ICEs were balanced between treatment arms, and the properties of the chosen estimation methods. For example, given the low-and-middle-income-countries settings of active TB trials, loss to follow-up and certain ICEs such as and treatment interruptions can be more common than in high-income settings. The approaches to handling the ICEs could then have more significant consequences on how the regimens are evaluated. These are important questions, but are beyond the scope of this article.

### Limitations

While we have focussed on only two trials as our illustrative examples, we have knowledge of other trials whose outcomes were defined using similar approaches. We have provided general recommendations for how certain types of ICE could be handled, but what constitutes an ICE and appropriate strategies for handling the ICEs should be considered specifically in the context of each trial.

We focussed on considerations for handling ICEs as part of defining estimands. In practice, clearly estimation is an important consideration when choosing an estimand, and reliable estimation methods need to be identified before the choice of estimand is finalised. There is an emerging literature on the importance of choosing estimators that align with the estimands.^33^ In the context of TB treatment trials, further work is needed to identify practical estimation methods that align well with the chosen estimands.

We have chosen to consider perspectives of the patient and healthcare provider when evaluating the programmatic effect of new TB treatments. These are the two most important and relevant stakeholders to us, since our Unit aims to conduct trials that have a clear real-world impact in terms of improving standards of care for patients in practice. Other perspectives, for example, of the drug developer or regulator, may require additional estimands to be constructed, potentially with different strategies for handling the same ICEs. Although the patient perspective discussed in this article has not benefitted from input from patient representatives regarding what they would consider a poor outcome, we are doing separate work on patient-focussed outcomes for future trial designs. We recognise this as a limitation, but hope that the points raised here provide a useful starting point for input from patient representatives and community groups.

### Summary of recommendations

We propose the following considerations in defining the outcomes for TB trials. First, changes to allocated regimens should not necessarily be viewed as an unfavourable outcome. From the patient perspective, the potential harms associated with a change in the regimen should instead be directly quantified. Second, handling poor adherence using a per-protocol analysis makes sense from the paradigm of non-inferiority trials, but it can imply an unrealistic estimand in TB trials. Since per-protocol, as defined in recent TB trials, does not target a clear estimand, even if non-inferiority is demonstrated it does not necessarily mean that the intervention really is non-inferior for a reasonable estimand/clinical question. Future work should develop ways to estimate effects of the regimens that match programmatic settings more closely. Third, if the clinical question of interest is whether the patient is cured of any TB following treatment, then the treatment policy strategy could be used to handle re-infection with a different strain of TB. From a healthcare provider perspective, a hypothetical strategy could be used to evaluate the effect of treatment in a scenario where re-infection did not occur. Fourth, separating death, where possible, into TB-related and non-TB-related is helpful in defining estimands. Finally, loss to follow-up before the end of the trial should not always be regarded as an unfavourable outcome in itself, but loss to follow-up having not completed the assigned treatment may be considered as an unfavourable outcome.

## Conclusion

The estimand framework aims to give clarity to the different issues related to defining outcomes in TB trials and a common language for discussion and comparison across studies. The framework also challenges trialists to justify and improve their outcome definitions. By providing examples and rationale for how common ICEs could be handled in the context of the trials considered, we hope that this article will stimulate and inform discussions among trial teams on how estimands should be defined for their trials, including identifying potential ICEs, choosing appropriate strategies for handling them that are aligned with the research question, and using an analysis method consistent with that. Future TB trialists should consider all the above points in defining their estimands.
